# Repositioning of Tyrosine Kinase Inhibitors as Antagonists of ATP-Binding Cassette Transporters in Anticancer Drug Resistance

**DOI:** 10.3390/cancers6041925

**Published:** 2014-09-29

**Authors:** Yi-Jun Wang, Yun-Kai Zhang, Rishil J. Kathawala, Zhe-Sheng Chen

**Affiliations:** Department of Pharmaceutical Sciences, College of Pharmacy and Health Sciences, St. John’s University, Queens, NY 11439, USA; E-Mails: yijun.wang11@stjohns.edu (Y.-J.W.); yunkai.zhang12@stjohns.edu (Y.-K.Z.); rishil.kathawala10@my.stjohns.edu (R.J.K.)

**Keywords:** multidrug resistance, ABC transporters, tyrosine kinase inhibitor, clinical relevance, pharmacogenomics

## Abstract

The phenomenon of multidrug resistance (MDR) has attenuated the efficacy of anticancer drugs and the possibility of successful cancer chemotherapy. ATP-binding cassette (ABC) transporters play an essential role in mediating MDR in cancer cells by increasing efflux of drugs from cancer cells, hence reducing the intracellular accumulation of chemotherapeutic drugs. Interestingly, small-molecule tyrosine kinase inhibitors (TKIs), such as AST1306, lapatinib, linsitinib, masitinib, motesanib, nilotinib, telatinib and WHI-P154, have been found to have the capability to overcome anticancer drug resistance by inhibiting ABC transporters in recent years. This review will focus on some of the latest and clinical developments with ABC transporters, TKIs and anticancer drug resistance.

## 1. Introduction

Cancer, also known as malignant neoplasm or tumor, is the second most leading cause of death after cardiovascular diseases in United States and developing countries. Cancer is not a single disease, but rather it consists of around two hundred potent, heterogeneous diseases, which originate in specific organs such as the lung, breast, colorectum and prostate [1]. Cancer treatment is often consisted of surgery, radiation therapy, chemotherapy and combination therapy. Chemotherapy has been the first-line treatment of most cancers for several decades, and uses drugs with different chemical structures and different mechanisms of action. Chronic treatment of cancer with chemotherapeutic drugs produces resistance to that particular drug and reduces the effectiveness of anticancer agents, which enhances the possibility of “cancer recurrence” [2]. Anticancer drug resistance appears to be the leading obstacle in the process of chemotherapy. The most common mechanisms that produce drug resistance in cancer cells include: (1) altered cell cycle check points; (2) induction of emergency response genes; (3) alterations in membrane lipids; (4) compartmentalization; (5) inhibition of apoptosis; (6) altered drug targets; (7) decreased uptake and (8) increased efflux of drugs by ATP-binding cassette (ABC) transporters [3]. Since ABC transporter-mediated multidrug resistance (MDR) is the most aggressive and lethal form of drug resistance, it will be discussed here.

## 2. MDR and ABC Transporters

### 2.1. MDR

MDR is a phenomenon where cancer cells become resistant to drugs with different chemical structures and different mechanisms of action [4]. MDR in cancer chemotherapy is similar to resistance to antibiotics, which results in poor absorption, enhanced metabolism, environmental alterations, and poor penetration to specific sites, hence limiting drug delivery. MDR in cancer cells usually develops after repeated exposure to chemotherapeutic drugs for a long time, perhaps resulting from the overexpression of certain transporters that function to efflux drugs out of cells [2].

The mechanism of anticancer drug resistance is sophisticated, since the resistance can be produced by host factors (acquired) or genetic changes in cancer cells [5]. It consists of changes in the permeability of lipid bilayer membrane, suppression of apoptosis, upregulated DNA repair of cancer cells, inactivation or detoxification of drugs, alterations in the number of membrane receptors or transporters involved in accumulating or effluxing drugs from cells [6]. One of most common mechanisms that produce MDR in cancer cells is the overexpression of a family of specific transmembrane, energy-dependent transporters known as ATP-binding cassette (ABC) transporters. The ABC transporter family is the most abundant transmembrane protein family encoded in the human genome [7].

### 2.2. ABC Transporters

**Figure 1:**
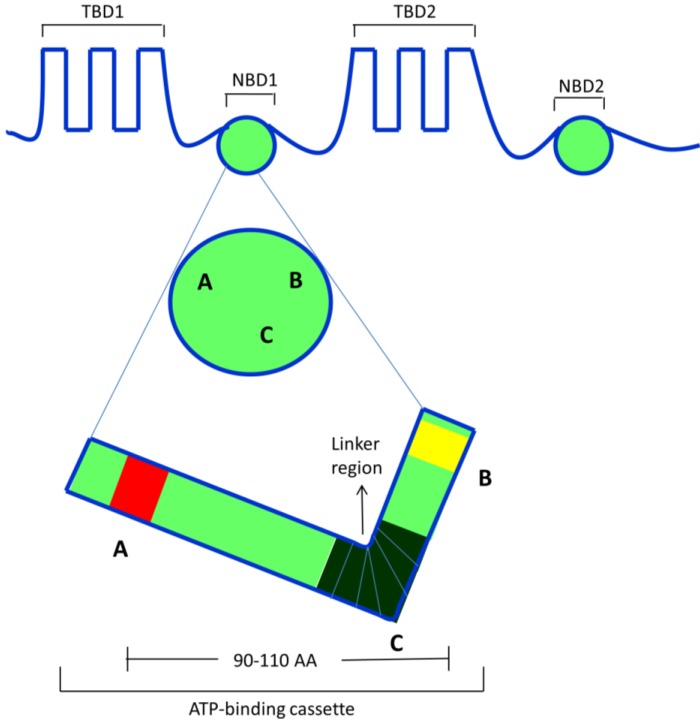
ATP-binding Cassette. ABC family members share this conserved consensus sequence of Walker A and B along with the C (signature or linker region). The ABC cassette is involved in effluxing the drugs out of the cells. NBD—nucleotide binding domain; TMD—transmembrane binding domain.

ABC transporters are a group of active transporter proteins that have various functions and ubiquitous presence in both prokaryotes and eukaryotes [8]. These transporters utilize energy derived from the hydrolysis of ATP to adenosine diphosphate (ADP) to transport their substrates across the membrane against a concentration gradient [9]. The term, ABC transporter, is derived from a conserved consensus sequence of 90–110 amino acids, which is possessed by most of the members of ABC family from yeast, bacteria to human beings [10]. The consensus contains Walker A and B motifs as well as the C motif (also named as signature or linker region), found upstream to Walker B motif joining Walker A and B motifs (Figure 1). The Walker A and B motifs play a significant role in the hydrolysis of ATP to ADP + Pi and energy coupling [11]. Up to now, 49 members of the ABC transporter family have been isolated and identified (even though some of the literature only recognizes 48 members, since ABCC13/MRP10 is a nonfunctional gene encoding transporter). Structurally, ABC transporters have two nucleotide binding domains (NBDs) and two transmembrane binding domains (TMDs) [12]. The NBDs contains conserved ABC that is responsible for binding and extruding physiological and xenobiotic substrates out of cells. The NBDs hydrolyze ATP via ATPase enzyme and play an essential role in conferring MDR to a variety of chemotherapeutic drugs [13]. ABC transporter family can be divided into seven subfamilies ABC-A to G, which are further subdivided into subfamilies (except ABCE/OABP family) depending on their structure similarity or difference in their TMDs [14].

### 2.3. ABCB1/P-Glycoprotein (P-gp/MDR1)

ABCB1 or P-gp, an encoded gene product of human *MDR1* (multidrug resistance 1) with a molecular weight of 160–170 kDa, is the first discovered ATP-dependent system [15,16]. The ABCB1 is an apical plasma membrane transporter that is ubiquitously expressed in kidneys, intestine, placenta, liver, adrenal glands and blood-brain barrier (BBB) cells, where it normally functions to extrude certain xenobiotics and protect the cells from toxicants [17,18]. The overexpression of ABCB1 has been shown to produce a primary effect in MDR to the chemotherapy of cancer and confer significant resistance to a wide variety of anticancer substrate drugs, such as anthracyclines, vinca alkaloids, taxanes, epipodophyllotoxins, imatinib mesylate and so on [14,18]. Considerable evidence suggested that there are strong relationships between the overexpression of ABCB1 and various cancers, like advanced gastrointestinal stromal tumor (GIST), non-small cell lung cancer (NSCLC), fallopian tube, ovarian and thyroid cancer [19,20,21,22,23]. Interestingly, the absence of ABCB1 expression in some MDR cells has triggered more studies on other important transporters with efflux functions, such as ABCC subfamily and ABCG2. More and more studies are being performed to get a better understanding about the role of ABC transporters in MDR in cancer treatment.

### 2.4. ABCC/Multidrug Resistance Proteins (MRPs)

ABCC subfamily, commonly called as multidrug resistance protein (MRP) family, has been shown to be associated with MDR in various cancers, including lung cancer (both small and non-small cell lung cancers), bladder cancer and breast cancer [24]. There are 13 members in ABCC subfamily (ABCC1 to ABCC13), in which ABCC13/MRP10 is a nonfunctional gene encoding transporter. The MRP family can be further classified into two groups on the basis of their structural topology. One group including ABCC1/MRP1, ABCC2/MRP2, ABCC3/MRP3, ABCC6/MRP6 and ABCC10/MRP7, has three TMDs and two NBDs, and the other group including ABCC4/MRP4, ABCC5/MRP5, ABCC11/MRP8 and ABCC12/MRP9, has two TMDs and two NBDs [25]. The substrate profile of ABCC subfamily transporters overlaps that of ABCB1 substrate list, but with a few exceptions such as taxanes (paclitaxel, docetaxel), which are poor substrates of most of the ABCC family members except ABCC10. ABCC has high affinity for negatively charged hydrophobic drugs; otherwise ABCB1 prefers to transport neutral or positive charged hydrophobic drugs [26]. The ABCC subfamily members are involved in the transport of compounds formed after phase II metabolism, like glutathione sulfate or glucuronide conjugation, and other organic anions, like methotrexate (MTX). Therefore, they are also called as multispecific organic anion transporters (MOAT) [27]. Mutations or absence of ABC transporters can trigger some specific diseases. For instances, mutations of *ABCC2/MRP2/cMOAT* gene would lead to mild liver disease associated with conjugated hyperbilirubinamia, also called Dubin-Johnson Syndrome [28]. In addition, mutations within the *ABCC6/MRP6* gene would cause a condition known as Pseudoxanthoma Elasticum Disorder, which is characterized by calcification of elastic fibers of skin, retina and arteries forming lesions [29].

With the help of reverse transcription-PCR analysis, Hopper *et al*. reported a low level of *ABCC10* transcript expression in the skin, testes, spleen, stomach, colon, kidney, heart and brain [30]. However, *ABCC10* transcripts were difficult to detect by Northern blot analysis, indicating that it has a low level of expression in many tissues. It has been shown that *ABCC10* transcript expression takes place (from highest to lowest) in the pancreas, then liver, placenta, lungs, kidneys, brain, ovaries, lymph nodes, spleen, heart, leukocytes and colon [31]. The transfection of HEK293 cells with the *ABCC10* gene confers resistance to various anticancer drugs including docetaxel, paclitaxel, vincristine, vinblastine, cytarabine, gemcitabine, epothilone B [32]. Chen *et al*. reported that ABCC10 can transport LTC4 while it does not transport glycocholic acid, taurocholic acid, methotrexate, folic acid, cAMP or cGMP, which are substrates for other MRP family members [33]. It has been found that ABCC10 has associations with vinorelbine and paclitaxel resistance in non-small cell lung cancer [34]. ABCC10 is also present in salivary gland adenocarcinoma [35]. In addition, *ABCC10* transcripts have been detected in HepG2 liver cancer cell line and two prostate cancer cell lines (CWR22Rv1 and TSU-PR1) [36].

### 2.5. ABCG2/Breast Cancer Resistance Protein (BCRP)/Mitoxantrone Resistant Protein (MXR)

The ABCG2 protein is a 655-amino acid polypeptide and has a molecular weight of 72 kDa. Because of containing only one TMD and one NBD, ABCG2 is the first half transporter in the ABC transporter family, which plays an essential role in regulating MDR in cancer cells. It has been indicated that ABCG2 would have to dimerize with itself (homodimerize) or other members of ABCG subfamily (heterodimerize) to have the capacity of performing ABC transporter like effluxing functions [37]. ABCG2 was first discovered in breast cancer cell line MCF-7, where it became resistant to DOX (MCF-7/AdrVp). Therefore, it is also popularly known as breast cancer resistant protein (BCRP) [38]. It has been found in mitoxantrone (MX) selected colon cancer cell line S1-M1-80, hence giving ABCG2 the name of mitoxantrone resistant protein (MXR) [39]. The wide substrate profile of ABCG2 is comprised of organic anion conjugates, nucleoside analogous, organic dyes, tyrosine kinase inhibitors (TKIs), anthracyclines (such as DOX, MX), camptothecin-derived indolocarbazole topoisomerase I inhibitors, MTX, and flavopiridols [6]. The ABCG2 transporter is a modulator of MDR in different kinds of caners, like breast, colon, gastric, small cell lung, ovarian, intestinal cancers and melanomas [12].

ABCG2 is a widely distributed transporter that is present mainly in the plasma membrane. The high expression levels of ABCG2 protein are found in the liver canalicular membrane, at the luminal surface of the endothelial cells of human brain microvessels, in the apical membrane of the epithelium of the small intestine, and in the placental syncytiotrophoblasts. Moreover, the relatively lower expression of ABCG2 is found in the colon, ovary veins, kidney, adrenal, lung, prostate, heart, stomach, spleen, cervix, breast ducts and lobules [40,41]. This strategic and systematic distribution reveals that ABCG2 plays a significant role in protecting the fetus and adults against toxins and xenobiotics [42]. For instance, the expression of ABCG2 has the potential to regulate the concentrations of fluoroquinolones in the breast milk [43]. Being in a situation of hypoxia, increasing expression of ABCG2 prevents accumulation of heme and protects mitochondrial from death [44]. The ABCG2 transporter plays a significant pharmacokinetic role in absorption, distribution, metabolism, and elimination (ADME) of drugs that are ABCG2 substrates, and regulating the drug bioavailability [6].

**Figure 2:**
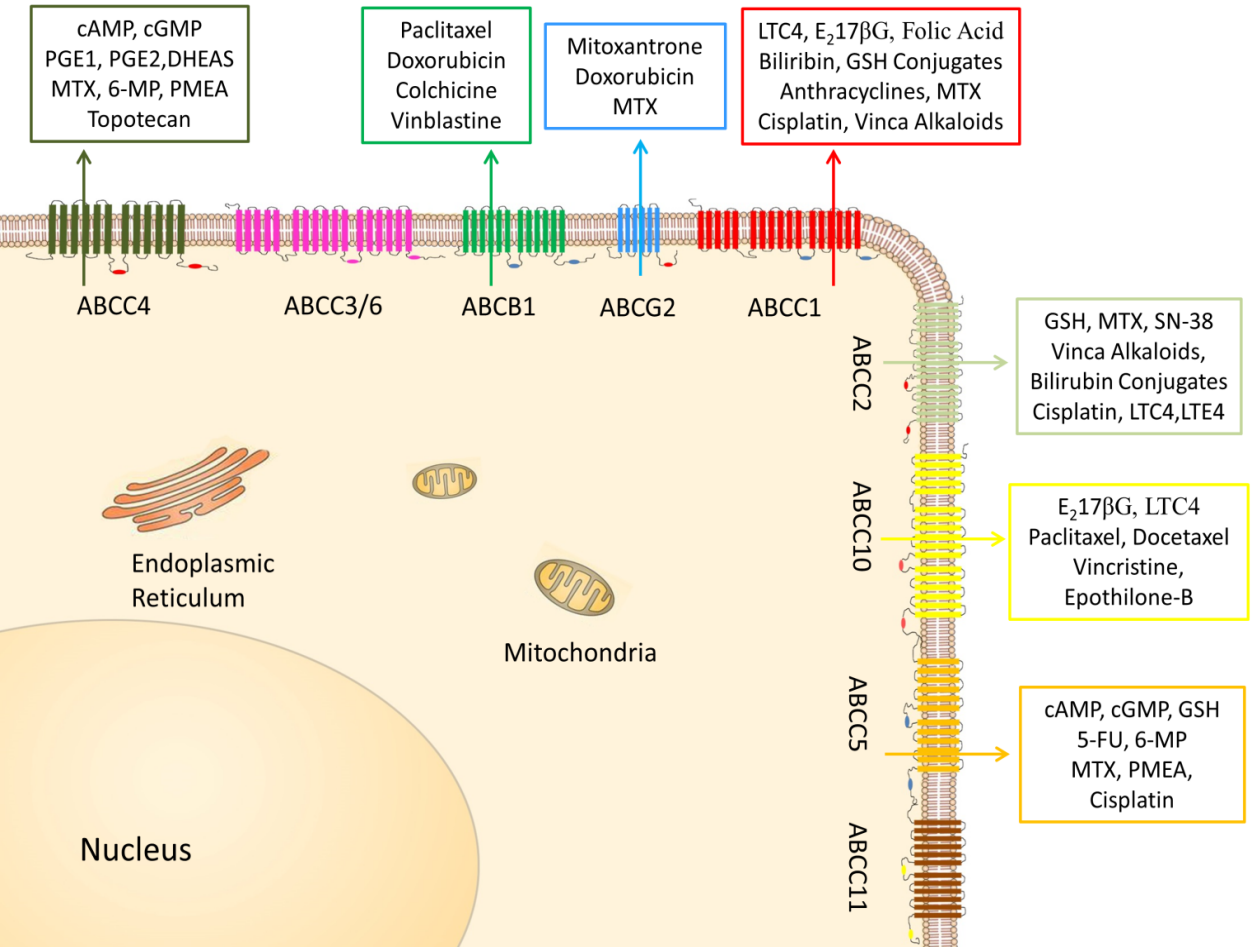
The topography and localization of ABCB1, ABCG2, short-(ABCC4, ABCC5, ABCC11) and long-(ABCC1, ABCC2, ABCC3, ABCC6, ABCC10) forms of ABCCs. Each of these ABC transporters pumps out a variety of endogenous and xenobiotic substrates. These transporters are mainly distributed apically or basolaterally. 5-FU: 5-fluorouracil; 6-MP: 6-mercaptopurine; DOX: doxorubicin; DHEAS: dehydroepiandrosterone; DNP: dinitrophenol; E_2_17βG: -estradiol-17β-D-glucuronide; GSH: glutathione; LTC4: leukotriene C4; MTX: methotrexate; PMEA: 9-(2-phosphonylmethoxyethyl)adenine; SN-38: 7-ethyl-10-hydroxycamptothecin.

It is the overexpression of ABCG2 in a variety of cell lines and tissues that becomes an essential MDR factor in solid tumors, such as breast cancer, colon cancer, gastric carcinomas, hepatocellular carcinoma, endometrial carcinoma, small cell lung cancer, melanoma, ovarian, gastric, and intestinal cancers [44]. High levels of ABCG2 expression were also present in acute lymphoblastic leukemia (ALL) and acute myelogenous leukemia (AML) [45]. Below is a figure illustrating some of the substrates for the ABC transporters (Figure 2).

## 3. Reversal of MDR in Cancers

### 3.1. Strategies for Overcoming MDR in Cancer Cells

Successful chemotherapy via using chemotheraperutic drugs can only be accomplished on condition that optimal pharmacokinetics, tumor penetration, and intracellular concentrations are conserved inside the cancer cells. However, as described earlier, overexpression of ABC transporters produces MDR and hinders effective cancer chemotherapy development by extruding drugs outside the cell via ABC transporters. Hence, it is essential to develop modulators of ABC transporters that have the potential to block or inactivate them, in order to increase the concentration of anti-cancer drugs within the cells. A number of strategies to overcome ABC transporter mediated MDR have been developed.

Some other strategies to combat MDR have been put forward, such as drug nanoencapsulation for effective delivery and better bioavailability, biological antibodies against ABCB1 (*i.e.*, MRK16), and some highly specific peptide analogues of TMDs, which probably impede the function or appropriate assembly of target ABC transporter [46]. Application of medical nanotechnology method for transporting anticancer drugs to the sites of solid tumors after systemic administration brings new opportunities for optimal drug delivery. The small size and enlarged surface area of nanoparticles produce improved solubility and stability of anticancer drugs, leading to a corresponding improvement in prevention, detection and treatment of drug-resistant cancer cells with minimal toxicity towards normal cells [47,48]. Controlled release, prolonged circulation, excellent ability to target the sites of action and bypass drug resistance mechanisms are some of advantages that the application of medical nanotechnology brings [49]. Furthermore, molecular strategies by using hammerhead ribozymes against ABCG2, antisense oligonucleotides and small interference RNA (siRNA) have also been proposed [50]. Even though all these strategies showed efficacy *in vitro* studies, their clinical applications are limited because of the shortness in delivery, stability and potency. While making efforts to establish a profile of substrates and inhibitors for ABC transporters involved in MDR, some inhibitors showed positive correlation that potentiated the toxicity of other chemotherapeutic drugs in the presence of ABC transporter. For instance, thiosemicarbazone (NSC73306) showed higher toxicity in which ABCB1 protein is overexpressed [51]. If so, we probably can also make well use of overexpression of ABC transporters to overcome MDR in cancer cells.

### 3.2. TKIs as Modulators for Anticancer Drug Resistance

**Figure 3:**
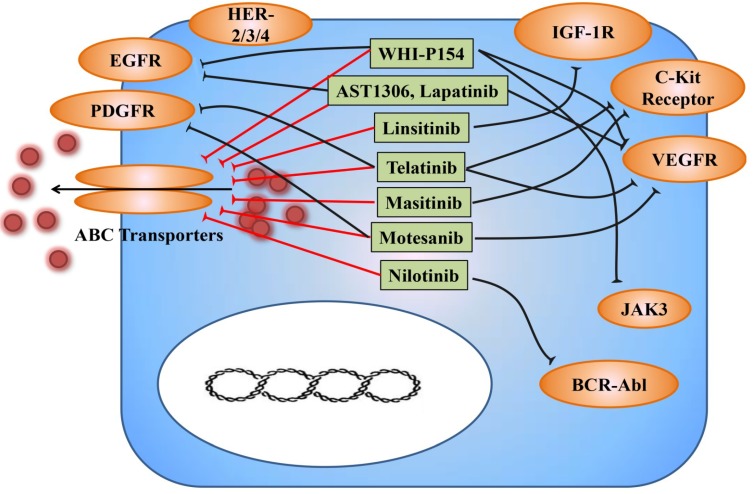
Different types of small-molecule tyrosine kinase inhibitors (TKIs) antagonize MDR mediated by ABC transporters. Overexpression of ABC transporters leads to accelerated efflux of anticancer drugs from cancer cells. Modulation of ABC transporters by different types of TKIs, such as AST1306, lapatinib, linsitinib, masitinib, motesanib, nilotinib, telatinib and WHI-P154, leads to enhanced intracellular accumulation of anticancer drugs. BCR-Abl, breakpoint cluster region-Abelson; JAK3, Janus kinase 3; VEGFR, vascular endothelial growth factor receptor; c-Kit, mast/stem cell growth factor receptor; IGF-1R, insulin-like growth factor 1 receptor; EGFR, epidermal growth factor receptor; PDGFR, platelet-derived growth factor receptor.

Tyrosine kinase inhibitors (TKIs) are characterized as inhibitors that can block the phosphorylation mediated by tyrosine kinase. TKIs may interfere with kinases via different mechanisms such as blocking the binding of ATP, blocking allosteric sites or inhibiting receptor internalization [52]. Suppression of tyrosine kinases can disrupt several cellular functions and then lead to decreased cell proliferation and decreased apoptosis [53]. Interestingly, TKIs have been found to have the capability to overcome anticancer drug resistance in recent years. For example, vemurafenib, a specific B-Raf inhibitor is used for treatment of metastatic melanomas [54]. It has been found that vemurafenib could reverse the ABCB1, ABCC10 and ABCG2-mediated MDR. It enhanced the intracellular accumulation of paclitaxel in ABCB1 and ABCC10 overexpressing cells and and mitoxantrone in ABCG2 overexpressing. In contrast, no significant change in the expression levels of ABCB1, ABCG2 and ABCG2 was observed when these cells were exposed to 20 μM vemurafenib for 96 h [55]. Furthermore, we discovered that TKIs mainly reglulated MDR by targeting at ABC transporters as substrates or modulators [56,57,58,59,60,61,62,63,64,65] (Figure 3) (Table 1). Here, we will focus on the effects of small-molecule TKIs on anticancer drug resistance especially by interfering with ABC transporters.

ATPase studies on selected ABC transporters are performed to illustrate these TKIs stimulate ATPase activity. Therefore, these TKIs may sever as competitive inhibitors of ABC transporters. However, although high possibility exists, ATPase evidence itself may not sufficient for proving of competitive inhibition mode, therefore it is not included in Table 1. Tyrosine kinase inhibitors mentioned in the manuscript are basically hydrophobic compounds; their calculated logP value usually ranges from 3 to 6. Hydrophobic character is essential for ABC transporters inhibition. The drug binding sites in the transmembrane domain and ATP binding domain of ABC transporters are both usually highly hydrophobic in nature, such as ABCB1 based on its structure [66]. The contribution of hydrophobic compounds may be explained as their ability to distribute within the biomembrane where ABC transporters locate. Moreover, for the pharmacophoric views, these TKIs have several essential features such as hydrophobic groups and/or aromatic ring centers, hydrogen bond acceptors and some TKIs have positively charged ionizable group. These features have been described for ABCB1 inhibition [67,68,69]. The TKIs as antagonists for ABC transporters are discussed below.

#### 3.2.1. AST1306

AST1306 is a novel multi-targeted TKI that inhibits EGFR and ErbB2 [70,71]. Design of AST1306 was based on combination of chemical structure of lapatinib and key chemical group of irreversible EGFR inhibition [70]. AST1306, at concentration of 1 μM, was about to overcome multidrug resistance mediated by ABCG2. The reversal effect was not mediated through reducing the expression level of ABCG2 transporter. Furthermore, the intracellular level of [^3^H]-MX in ABCG2-overexpressing cells was significantly increased by AST1306. In the meantime, [^3^H]-MX efflux was accelerated by incubation with AST1306. As reported, AST1306 stimulated ATP hydrolysis by ABCG2 therefore might interact with ABCG2 as a substrate [63]. Overall, AST1306 might broaden the usage of TKIs as novel reversal agent of ABCG2 transporter by increasing clinical response in combined chemotherapy.

**Table 1:** Tyrosine kinase inhibitors (TKIs) as modulators of ABC transporter.

Compound	Concentrations (μM)	Targets	Targeted ABC Transporters	Effects on Targeted ABC Transporters Substrate	Effects on Targeted ABC Transporters Protein Expression
AST1306	0.25, 1	EGFR and ErbB2	ABCG2	+	↔
Lapatinib	0.625, 1.25, 2.5	EGFR, HER-2, HER-3 and HER-4	ABCB1 and ABCG2	+	↔
Linsitinib (OSI-906)	1, 2	IGF-1R	ABCG2 and ABCC10	+	↔
Masitinib	1.25, 2.5	c-Kit	ABCG2 and ABCC10	+	↔
Motesanib (AMG-706)	1, 3	VEGFR-1/2/3, PDGFR and c-Kit	ABCB1 and ABCG2	+	↔
Nilotinib (AMN-107)	1, 2.5	BCR-Abl	ABCB1, ABCG2 and ABCC10	+	↔
Telatinib	0.25, 0.5, 1	VEGFR-2/3,PDGFR-β	ABCG2	+	↔
WHI-P154	1, 4	JAK3, EGFR, VEGFR	ABCG2	+	↔

(+): this compound shows reversal activity on the targeted ABC transporters; (↔): no significant alterations.

#### 3.2.2. Lapatinib

The epidermal growth factor receptor (EGFR) and human epidermal receptor type 2, 3, 4 are often overexpressed and activate a series of signaling pathways in human tumors [72]. Lapatinib is an orally active tyrosine kinase inhibitor of Her-2 and EGFR. As lapatinib acts at ATP-binding site, it may also inhibit the function of ABC transporters and therefore interrupt MDR. Following study revealed that lapatinib sensitize ABCB1- and ABCG2-overexpressing MDR cells in concentration-dependent pattern. Lapatinib also enhanced the accumulation of chemotherapeutic agents via blockage of ABC transporters. Dai *et al*. also demonstrated that lapatinib might be a substrate of ABCB1 and ABCG2 by conducting ATPase assays [56]. The *in vivo* studies further confirmed the reversal effect of lapatinib on ABCB1-mediated MDR, therefore indicated that combination of lapatinib with other anticancer drugs may be important in surmounting clinical resistance in cancer chemotherapy [56].

#### 3.2.3. Linsitinib (OSI-906)

Linsitinib (OSI-906) is a selective inhibitor of insulin-like growth factor 1 (IGF-1R)/insulin receptor (IR), and it inhibits IGF-1R auto phosphorylation and activation of the downstream signaling protein Akt, ERK1/2 and S6 kinase [73]. Therefore, linsitinib inhibits tumor growth in IGF-1R-driven xenograft mouse model [74]. Recent study of linsitinib investigated its reversal effect on MDR mediated by ABC transporters. Linsitinib was shown to overcome MDR by significantly potentiating the effect of anti-neoplastic drugs mitoxantrone, SN-38 in ABCG2-overexpressing cells, and the effect of paclitaxel, docetaxel, vinblastine in ABCC10-overexpressing cells [62]. They further concluded that linsitinib attenuated ABCG2- and ABCC10-mediated MDR by inhibiting their function instead of altering protein expression via several radioactive labeled or immune hybridation studies [62]. Their findings suggested that linsitinib might be beneficial to the traditional chemotherapy by overcoming MDR as a supplement agent.

#### 3.2.4. Masitinib

Masitinib is a novel pheylaminothiazole derivative TKI used for gastrointestinal stromal tumor and pancreatic cancer [75,76,77]. Recently, Kathawala *et al*. discovered that masitinib could antagonize ABCG2-mediated multidrug resistance [78]. Masitinib (1.25 and 2.5 μM) significantly reversed resistance to mitoxantrone (MX), SN-38 and doxorubicin in ABCG2 overexpressing cell lines. Subsequently, it has been reported that masitinib could reverse ABCC10-mediated MDR [60]. Western blotting analysis indicated that no significant alteration in the expression levels of ABCG2 and ABCC10 was observed when ABCG2 and ABCC10 overexpressing cells were exposed to 2.5 μM mastinib for 72 h. Moreover, *in vivo* study indicated that the sizes of ABCC10-expressing tumors in nude athymic mice significantly blunted by the combination treatment of paclitaxel and masitinib [60]. If these effects can be clinically translated, they suggested that masitinib might be efficacious in treating MDR cancers in combination with paclitaxel or mitoxantrone.

#### 3.2.5. Motesanib (AMG-706)

Motesanib, a novel nicotinamide derivative, was identified as a potent, orally bioavailable inhibitor of the vascular endothelial growth factor receptor 1 (VEGFR1/Flt1), VEGFR2/kinase domain receptor/Flk-1, VEGFR3/Flt4, platelet-derived growth factor receptor (PDGFR) and Kit receptors in preclinical models [79]. Recent studies reported that motesanib significantly sensitized both ABCB1-transfected and drug-selected cell lines overexpressing this transporter to its substrate anticancer drugs. Motesanib significantly increased the accumulation of [^3^H]-paclitaxel in ABCB1 overexpressing cells by blocking the efflux function of ABCB1 transporter. In contrast, no significant change in the expression levels and localization pattern of ABCB1 was observed when ABCB1 overexpressing cells were exposed to 3 μM motesanib for 72 h [65]. Furthermore, the stimulatory effect of motesanib on the ATPase activity of ABCB1 revealed the direct contact between the drug and the transporter. In congruence with these findings, the docking studies suggested preferable binding of motesanib inside the transmembrane zone of homology modeled human ABCB1. In addition, motesanib could also moderately reverse ABCG2-mediated MDR [65]. These findings may be useful for cancer combination therapy with TKIs in the clinic.

#### 3.2.6. Nilotinib (AMN-107)

Chronic myeloid leukemia (CML) is often associated with abnormal *BCR-Abl* gene and TK dysfunction [80]. Nilotinib, a BCR-Abl tyrosine kinase inhibitor (TKI), was developed to surmount resistance or intolerance to imatinib in patients with Philadelphia positive chronic myelogenous leukemia [81]. Previous studies reported that nilotinib significantly reversed ABCB1- and ABCG2-mediated MDR *in vitro* [58]. Afterwards, it has also been reported that nilotinib could reverse ABCC10-mediated paclitaxel resistance in transfected HEK cell lines [57]. Furthermore, recent *in vivo* study from our laboratory demonstrated that nilotinib could strengthen the anticancer activity in ABCB1-, ABCG2, and ABCC10-xenograft MDR models in athymic mice [59]. Combination treatments of nilotinib with paclitaxel or doxorubicin significantly decrease MDR tumor sizes without apparent toxicity or weight loss. Separate PK study also suggested that nilotinib may increase plasma AUC of paclitaxel [59]. Importantly, MDR-mediated insensitivity in the clinic might be new target of nilotinib due to its positive actions.

#### 3.2.7. Telatinib

Telatinib is an effective small-molecule TKI of the vascular endothelial growth factor receptor (VEGFR-2/VEGFR-3), platelet-derived growth factor receptor beta (PDGFR-β) and c-Kit [82]. Recent study demonstrated that telatinib could reverse ABCG2-mediated MDR *in vitro*, and this reversal activity also persisted *in vivo* [61]. Telatinib (15 mg/kg) with doxorubicin (1.8 mg/kg) significantly decreased the growth rate and size of ABCG2 overexpressing tumors in a xenograft nude mouse model [61]. Combination of telatinib with specific ABCG2 substrate drugs may be useful in treating human carcinomas especially those overexpress ABCG2 in clinic.

#### 3.2.8. WHI-P154

WHI-P154 is a synthesized dimethoxyquinazoline compound that has potent inhibitory activity against JAK3, EGFR, VEGFR and so on [83,84]. Besides its tyrosine kinase inhibition, recent report discovered that WHI-P154 could significantly enhance the chemotherapeutic effect of anticancer agents in ABCG2 overexpressing cells. WHI-P154 could also partially reverse ABCC1-mediated MDR. WHI-P154 has been shown to enhance the sensitivity of ABCG2 overexpressing cells to two established ABCG2 substrates, MX and SN-38, in a dose-dependent pattern [64]. Moreover, intracellular accumulation of [^3^H]-MX in the ABCG2 overexpressing cell lines was significantly increased by WHI-P154. Zhang *et al*. further suggested WHI-P154 might competitively inhibit ABCG2 by serving as a substrate, proven by enhanced ATPase activity [64]. They suggested an outlook for WHI-P154 to improve chemotherapy in MDR patients.

### 3.3. Effect of ABC Transporters Inhibition by TKIs on the Pharmacokinetics and Toxicity of Concomitantly Administered Anticancer Drugs

It was found that the combination therapy of nilotinib and paclitaxel showed potent inhibitory effect on KB-C2 tumor growth (size and weight) compared to vehicle, nilotinib or paclitaxel treated groups. No apparent weight loss or phenotypic changes were observed among the treatment groups compared with animals treated with vehicle. Furthermore, the combination treatment did not cause increased toxicity, instead it uniformly improved the efficacy of paclitaxel and doxorubicin in the ABCB1, ABCG2 and ABCC10 MDR-xenograft models compared to paclitaxel or doxorubicin treatment alone [59]. In the pharmacokinetic study, the total tumor exposure to paclitaxel as indicated by AUC (area under the drug concentration-time curve) revealed a 33% elevation, 1141.6 h ng/mL in the nilotinib-paclitaxel combination group versus 764.5 h ng/mL in the paclitaxel alone group. The paclitaxel plasma AUC showed a 27% increase, 5838.2 h ng/mL in the combination group *versus* 4217.4 h ng/mL in the paclitaxel alone group. It has been suggested that nilotinib can influence the PK behavior of paclitaxel most likely due to its inhibition of ABCB1 transporter [59].

It has been reported that the masitinib-paclitaxel combination group significantly reduced the sizes, weights and tumor volumes of the tumors expressing the ABCC10 transporter as compared to the vehicle, masitinib or paclitaxel alone group [60]. Further pharmacokinetic data showed that coadministration of masitinib and paclitaxel produced a transient increase in the plasma levels of paclitaxel. The tumor exposure to paclitaxel was significantly enhanced, 69.93 ± 14.15 ng/mL in the masitnib-paclitaxel combination group versus 16.31 ± 6.45 ng/mL in the paclitaxel alone group. However, the mastinib-paclitaxel combination therapy did not significantly increase the concentration of paclitaxel in the lungs as compared to the paclitaxel alone treatment [60].

The inhibition of these drug efflux transporters could increase oral bioavailability or decrease biliary excretion resulting in higher systemic drug exposures producing more potent cell toxicity. Lapatinib and gefitinib have been reported to have the capability to strengthen the bioavailability of certain anticancer drugs [56,85]. It has been shown that gefitinib can reverse resistance to SN-38 (the active metabolites of irinotecan) in cells that overexpressing ABCG2 *in vitro* [86]. The pharmacokinetic study demonstrated that oral gefitinib co-administration did not lead to any significant alterations in clearance of i.v. administered irinotecan. Interestingly, gefitinib can highly enhance the oral bioavailability of irinotecan. It is suggested that gefitinib might reverse tumor resistance to SN-38 mediated by ABCG2 by blocking the drug efflux function and might be beneficial in humans to regulate the oral bioavailability of poorly absorbed drugs such as irinotecan [85].

## 4. Clinical Relevance of ABC Transporters

### 4.1. ABCB1

The clinical significance of ABCB1 antagonism has been studied as a potential therapeutic strategy since last few years. Nevertheless, it has proven to be quite sophisticated to establish the role of ABCB1 as a “target” to enhance chemotherapeutic effect of the anticancer drugs [87]. The major factors leading to unsuccessful clinical inhibition of ABCB1 include altered pharmacokinetics of chemotherapeutic drugs such as doxorubicin, vincristine, paclitaxel, daunorubicin and etoposide, both as a result of ABCB1 inhibition and as a result of inhibition of other drug transporters and CYP3A4 [88,89,90,91,92,93]. Higher mortality was observed in the AML patients treated with cyclosporine analogue valspodar (PSC-833) [93]. The most promising data for MDR modulation until now is the SWOG 9126 trial with using high dose cyclosporine infusion in relapsed and high-risk acute myeloid leukemia (AML) patients [94]. However, other Phase III studies in AML patients in the administration of cyclosporine or valspodar have been negative [93,95]. Diverse patient populations and the majority of secondary AML patients participating in SWOG 9021 showing high expression levels of ABCB1 could lead to contradictory results between these studies. Patient selection on the basis of screening for functional ABCB1 expression will be crucial for future studies. Recently, a phase III trial of the potent and specific ABCB1 inhibitor zosuquidar reported that this drug did not improve the outcome of older patients with newly diagnosed acute myeloid leukemia [96]. Incomplete inhibition of ABCB1 leads to drug-drug interactions and producing toxicities; suboptimal design of clinical trials eventually results in the failure of therapeutic approach to inhibit ABCB1. Thus, the most primary reason for this may be the existences of multiple and redundant mechanisms of drug resistance in human cancers. In this regard, relatively non-specific drugs such as cyclosporine may be of an advantage by inhibiting multiple drug transporters [97].

On the other hand, treatment of human cancers with histone deacetylase (HDAC) inhibitors may result in expression of ABCB1 or ABCG2 or both. Interestingly, FK228, a class I HDAC inhibitor, induced ABCB1 overexpression in normal peripheral blood mononuclear cells, in circulating tumor cells, acute promyelocytic leukemia cells and osteosarcoma [97,98]. Moreover, in acute promyelocytic leukemia cells the co-administration of all-trans retinoic acid with FK228 increased acetyl-H3-Lys9 and acetyl-H4 at the ABCB1 promoter region [99]. Hence, resulting in MDR from upregulation of ABCB1 by HDAC inhibitors such as FK228.

### 4.2. ABCG2

The presence of ABCG2 is linked with poor prognosis and clinical drug resistance in AML. Since ABCG2 is likely to be expressed in a small subset of acute myeloid leukemia cells with primitive characteristics, such as expression of CD34 but not CD38, the evaluation of ABCG2 and its inhibition should be focused on those cells [100,101]. Until recently, clinical significance of ABCG2 expression has not been established in acute lymphoblastic leukemia. It has been reported that ABCG2 expression is detected at a higher level in B lineage acute lymphoblastic leukemia than in T lineage acute lymphoblastic leukemia [102,103]. High expression of ABCG2 in premature chronic myeloid leukemia cell populations has been reported. Jordanides *et al.* detected 6.8-fold higher *BCRP* mRNA expression in CD34+ cells derived from chronic myeloid leukemia patients than normal CD34+ cells [104]. Sixty-seven cases of ABCG2 expression in diffuse large B-cell lymphoma (DLBCL) using immunohistochemistry are reported and it was found that ABCG2 expression levels were positively correlated with sonic hedgehog ligand expression and that patients with high ABCG2 expression showed significantly shorter overall survival (*p* = 0.031) and failure-free survival (*p* = 0.029) compared with patients with low or no expression of ABCG2 [105]. Szczuraszek *et al.* examined expression of cyclooxygenase-2 (COX-2) and ABC transporters in 56 previously non-treated patients by immunohistochemistry [106]. They found that elevated expression of ABCG2 was signature for the patients that didn’t respond to chemotherapy and for cases with shorter progression-free survival time in a 2.5 years follow-up and concluded that ABCG2 may be a crucial factor in primary resistance of non-Hodgkin’s lymphomas. In small cell lung cancer, Kim *et al.* investigated ABCG2 expression in tumor biopsy specimens obtained before chemotherapy from 130 patients who later received platinum-based combination chemotherapy, and found significant associations between ABCG2 expression and both response and progression-free survival [107]. On the other hand, Rijavec *et al.* investigated *ABCG2* mRNA expression by RT-PCR in 14 small cell lung cancer and seven non-small cell lung cancer samples [108]. They observed significantly decreased *ABCG2* expression in metastatic small cell lung cancer cells compared to non-small cell lung cancer.

### 4.3. ABCCs

Apart from ABCB1 and ABCG2, clinical studies have indicated that ABCCs plays an important role in developing resistance to several chemotherapeutic regimens. Recently, it has been reported that ABCC1 is a predictive marker of the highly aggressive subtypes of breast carcinoma [109]. A significant overexpression of *ABCC1* and *ABCC2* mRNA has been found in locally advanced and metastatic colorectal carcinoma [110]. High levels of *ABCC1* mRNA has been linked with resistance to vincristine, etoposide and cisplatin in human lung carcinoma cell lines [111,112]. In another study it has been demonstrated that ABCC1 overexpression confers resistance to doxorubicin in prostate cancer cell lines [113]. Clinically detectable *ABCC1* mRNA expression has been associated with poor response to chemotherapy in acute lymphoblastic leukemia but not in acute myeloid leukemia [114]. The ABCC2 transporter actively effluxes taxanes, vincristine, etoposide, doxorubicin, methotrexate, and also cisplatin [115,116,117]. The ABCC3 transporter is expressed in lung cancer cells [112] and is known to cause *in vitro* resistance to doxorubicin, epipodophyllotoxins, and methotrexate [118,119]. The ABCC4 and ABCC5 transporters efflux purine analogues such as 6-mercaptopurine and 6-thioguanine [26,120]. In addition, ABCC4 confers resistance to camptothecins analogues [121] whereas ABCC5 produces resistance to 5-fluorouracil and gemcitabine [122]. The ABCC6 gene conferred a low level of resistance to epipodophyllotoxins, certain anthracyclines, actinomycins and of very low levels to cisplatin [123].

Clinically, a significantly high level of ABCC10 expression has been found in non-small cell lung cancer and acute myeloid leukemia after chemotherapy, causing acquired resistance [124,125]. The transfection of HEK293 cells with the *ABCC10* gene confers resistance to various anticancer drugs including paclitaxel, docetaxel, vincristine, vinblastine, vinorelbine, cytarabine, gemcitabine, 2',3'-dideoxycytidine, 9-(2-phosphonylmethoxyethyl)adenine (PMEA) and epothilone B [60]. In addition, the toxic response to paclitaxel is increased in *Abcc10* knockout mice, suggesting that the ABCC10 transporter plays a critical role in protection against xenobiotic toxicity [32].

## 5. Pharmacogenomics of ABC Transporters

Pharmacogenomics is the interdiscipline of pharmacology and genomics involving the study of the influence of genetic factors on the pharmacodynamic and pharmacokinetic profile of a drug. A number of previous studies suggested that genetic differences in individuals play an important role in the efficacy and adverse effects produced by drugs. The pharmacogenomic correlation of paclitaxel and ABCB1 has been contrasting. The systemic elimination of paclitaxel occurs by hepatic metabolism involving the CYP450 enzymes CYP3A4 and CYP2C8 [126] and biliary elimination of paclitaxel occurs by ABCB1 [127]. Several single nucleotide polymorphisms (SNP) in the *ABCB1* gene, including G1199T/A and the linked G2677T/A (Ala893Ser/Thr), are associated with progression-free survival after paclitaxel treatment [128,129] and C3435T (Ile1145Ile, wobble) as well as with paclitaxel-mediated peripheral neuropathy and neutropenia [130]. However, in other studies, no significant correlation was found between the pharmacokinetics of paclitaxel and the *ABCB1* genotype [130,131,132,133]. In conclusion, there is no strong evidence indicating that genetic variations in *ABCB1* gene are associated with resistance to paclitaxel and altered pharmacokinetics. It has been hypothesized that *ABCB1* genetic variants may alter the therapeutic response to paclitaxel. However, to date, this hypothesis has not been verified [134].

The role of ABCC1 polymorphisms is of note in terms of *in vivo* and clinical drug resistance profiles or toxicity. In one interesting report there has been association of the Gly671Val polymorphism in ABCC1 with anthracycline-induced cardiotoxicity among non-Hodgkin’s lymphoma patients treated with doxorubicin [135]. The authors therefore concluded that these patients might show increased doxorubicin accumulation in the heart. Whether this association is indeed caused by a reduction in doxorubicin transport due to this amino acid variation remains to be confirmed. Another *in vivo* study investigated if ABCC1 SNPs or haplotypes had any effect on nelfinavir exposure in the white blood cells of patients receiving nelfinavir. In that case, the authors did not find any association between the identified SNPs in ABCC1 with nelfinavir effects in white blood cells [136]. Dubin-Johnson Syndrome (DJS) is a condition characterized by conjugated hyperbilirubinemia resulting from ABCC2 deficiency in humans [137]. These findings also strengthened further research examining the genetic determinants of expression, localization and function of ABCC2 in other organs, as well as the impact of such SNPs on drug levels or efficacy. It is, however, not known whether this loss of hepatic ABCC2-mediated transport has a major impact on the pharmacokinetics of drug substrates in humans [138]. This is likely due to the fact that DJS is a relatively rare disorder, and drug toxicity has not been a noted feature of this syndrome. Interestingly, it was identified that a very rare but naturally occurring ABCC2 variation caused impaired methotrexate elimination and was associated with enhanced methotrexate toxicity. They also showed that this A>G mutation at position 1271 (Arg412Gly) influenced methotrexate transport *in vitro* [139]. Therefore these findings indicate that several regions of the ABCC2 protein are involved in the binding and transport of methotrexate. Currently, there are no published data about the pharmacogenetic correlation between paclitaxel and ABCC10 transporter. However, it has been reported that two ABCC10 SNPs (rs9349256 and rs2125739) and their haplotypes are significantly correlated with kidney tubular dysfunction, resulting in accelerated efflux of tenofovir by ABCC10 [140]. In addition, it has been suggested that the ABCC10 SNP rs2125739 is correlated with nevirapine-induced hepatotoxicity [141], although this has been disputed [142].

Collectively, a large number of polymorphisms have been detected in ABCC1, ABCC2 and ABCC10, but only minority of them is located in coding regions. Even though the transporter expression, localization and function of ABCC1 and ABCC2 have been extensively studied, there are only very limited data that more convincingly suggest any clinical relevance of naturally occurring SNPs in ABCC1 ABCC2 and ABCC10.

Several reports suggesting that ABCG2 SNP analysis might be a useful strategy to predict systemic exposure to ABCG2 substrate drugs is becoming increasingly prevalent [143]. Of particular importance are recent studies that have demonstrated that subjects with a decreased ABCG2 activity owing to the Q141K variant are at an increased risk of gefitinib-induced diarrhea [144], and altered pharmacokinetics of 9-aminocamptothecin [145], diflomotecan [146], irinotecan [147], rosuvastatin [148], sulfasalazine [149,150] and topotecan [151]. However, contrasting results have been reported for other known ABCG2 substrates, such as doxorubicin [152], imatinib [153], nelfinavir [136] and pitavastatin [154]. Noticeably, several studies published by now contain small sample sizes in relation to the allelic and genotypic frequencies of the studied variants, as well as from a host of potentially confounding factors that influence their outcome. Most important among them are environmental and physiological factors that may affect expression of the transporter, and links to other genes or variants of putative relevance for drug absorption and disposition pathways. The inclusion of data on other variants in ABCG2 [143,155], and/or the use of haplotype profiles as opposed to testing unphased SNPs to predict certain phenotypes, may have clinical implications for agents such as erlotinib [156] and imatinib [157], but this remains to be clarified for most drugs. Furthermore, more detailed research into the influence of ethnicity on ABCG2 transporter function and expression in relation to substrate-specific phenotypes is urgently needed. The rs35605 SNP in ABCC1 have a significant impact on the pharmacokinetics and pharmacodynamics of irinotecan in advanced cancer patients [158]. Severe neurotoxicity has been significantly associated with ABCC1 SNP rs2074087 in colorectal cancer patients treated with adjuvant FOLFOX4 (5-fluorouracil, leucovorin and oxaliplatin) regimen [159].

## 6. Conclusions

Physiologically, the ABC transporters serve as a defense system, in which they pump out endogenous toxicants and xenobiotics such as antineoplastic drugs from cells. Therefore, it is essential to develop modulators of ABC transporters that could antagonize MDR by inhibiting the efflux activity of ABC transporters. In this way, the efficacy of anticancer drugs and the possibility of successful chemotherapy would be greatly increased. A number of strategies to combat ABC transporter-mediated MDR, such as application of TKIs, medical nanotechnology or pharmacogenomics, have been developed and could be very valuable in overcoming anticancer drug resistance.

## References

[1] Cockerill G.S., Lackey K.E. (2002). Small molecule inhibitors of the class 1 receptor tyrosine kinase family. Curr. Top. Med. Chem..

[2] Gottesman M.M. (2002). Mechanisms of cancer drug resistance. Annu. Rev. Med..

[3] Quintieri L., Fantin M., Vizler C. (2007). Identification of molecular determinants of tumor sensitivity and resistance to anticancer drugs. Adv. Exp. Med. Biol..

[4] Deeley R.G., Westlake C., Cole S.P. (2006). Transmembrane transport of endo- and xenobiotics by mammalian ATP-binding cassette multidrug resistance proteins. Physiol. Rev..

[5] Jemal A., Murray T., Ward E., Samuels A., Tiwari R.C., Ghafoor A., Feuer E.J., Thun M.J. (2005). Cancer statistics, 2005. CA Cancer. J. Clin..

[6] Mao Q., Unadkat J.D. (2005). Role of the breast cancer resistance protein (ABCG2) in drug transport. AAPS J..

[7] Ambudkar S.V., Kim I.W., Xia D., Sauna Z.E. (2006). The A-Loop, a novel conserved aromatic acid subdomain upstream of the walker a motif in ABC transporters, is critical for ATP binding. FEBS Lett..

[8] Wu C.P., Hsieh C.H., Wu Y.S. (2011). The emergence of drug transporter-mediated multidrug resistance to cancer chemotherapy. Mol. Pharm..

[9] Locher K.P., Borths E. (2004). ABC Transporter architecture and mechanism: Implications from the crystal structures of BtuCD and BtuF. FEBS Lett..

[10] Higgins C.F. (1992). ABC transporters: From microorganisms to man. Annu. Rev. Cell Biol..

[11] Hyde S.C., Emsley P., Hartshorn M.J., Mimmack M.M., Gileadi U., Pearce S.R., Gallagher M.P., Gill D.R., Hubbard R.E., Higgins C.F. (1990). Structural model of ATP-binding proteins associated with cystic fibrosis, multidrug resistance and bacterial transport. Nature.

[12] Tiwari A.K., Sodani K., Dai C.L., Ashby C.R., Chen Z.S. (2011). Revisiting the ABCs of multidrug resistance in cancer chemotherapy. Curr. Pharm. Biotechnol..

[13] Dean M. (2009). ABC transporters, drug resistance, and cancer stem cells. J. Mammary Gland Biol. Neoplasia.

[14] Dean M., Annilo T. (2005). Evolution of the ATP-binding cassette (ABC) transporter superfamily in vertebrates. Annu. Rev. Genomics Hum. Genet..

[15] Juliano R.L., Ling V. (1976). A surface glycoprotein modulating drug permeability in Chinese hamster ovary cell mutants. Biochim. Biophys. Acta.

[16] Schinkel A.H., Mol C.A., Wagenaar E., van Deemter L., Smit J.J., Borst P. (1995). Multidrug resistance and the role of P-Glycoprotein knockout mice. Eur. J. Cancer.

[17] Gottesman M.M., Fojo T., Bates S.E. (2002). Multidrug resistance in cancer: Role of ATP-dependent transporters. Nat. Rev. Cancer.

[18] Sarkadi B., Homolya L., Szakacs G., Varadi A. (2006). Human multidrug resistance ABCB and ABCG transporters: Participation in a chemoimmunity defense system. Physiol. Rev..

[19] Matsuo K., Eno M.L., Ahn E.H., Shahzad M.M., Im D.D., Rosenshein N.B., Sood A.K. (2011). Multidrug resistance gene (MDR-1) and risk of brain metastasis in epithelial ovarian, fallopian tube, and peritoneal cancer. Am. J. Clin. Oncol..

[20] Eechoute K., Sparreboom A., Burger H., Franke R.M., Schiavon G., Verweij J., Loos W.J., Wiemer E.A., Mathijssen R.H. (2011). Drug transporters and imatinib treatment: Implications for clinical practice. Clin. Cancer Res..

[21] Gao Y.J., Li B., Wu X.Y., Cui J., Han J.K. (2014). Thyroid tumor-initiating cells: Increasing evidence and opportunities for anticancer therapy (Review). Oncol. Rep..

[22] Gao B., Russell A., Beesley J., Chen X.Q., Healey S., Henderson M., Wong M., Emmanuel C., Galletta L., Johnatty S.E. (2014). Paclitaxel sensitivity in relation to ABCB1 expression, efflux and single nucleotide polymorphisms in ovarian cancer. Sci. Rep..

[23] Han J.Y., Lim H.S., Yoo Y.K., Shin E.S., Park Y.H., Lee S.Y., Lee J.E., Lee D.H., Kim H.T., Lee J.S. (2007). Associations of ABCB1, ABCC2, and ABCG2 polymorphisms with irinotecan-pharmacokinetics and clinical outcome in patients with advanced non-small cell lung cancer. Cancer.

[24] Leonard G.D., Fojo T., Bates S.E. (2003). The role of ABC transporters in clinical practice. Oncologist.

[25] Sodani K., Patel A., Kathawala R.J., Chen Z.S. (2012). Multidrug resistance associated proteins in multidrug resistance. Chin. J. Cancer.

[26] Kruh G.D., Belinsky M.G. (2003). The MRP family of drug efflux pumps. Oncogene.

[27] Norris M.D., Smith J., Tanabe K., Tobin P., Flemming C., Scheffer G.L., Wielinga P., Cohn S.L., London W.B., Marshall G.M. (2005). Expression of multidrug transporter MRP4/ABCC4 is a marker of poor prognosis in neuroblastoma and confers resistance to irinotecan *in vitro*. Mol. Cancer. Ther..

[28] Toh S., Wada M., Uchiumi T., Inokuchi A., Makino Y., Horie Y., Adachi Y., Sakisaka S., Kuwano M. (1999). Genomic structure of the canalicular multispecific organic anion-transporter gene (MRP2/cMOAT) and mutations in the ATP-binding-cassette region in Dubin-Johnson syndrome. Am. J. Hum. Genet..

[29] Ringpfeil F., Lebwohl M.G., Christiano A.M., Uitto J. (2000). Pseudoxanthoma elasticum: Mutations in the MRP6 gene encoding a transmembrane ATP-binding cassette (ABC) transporter. Proc. Natl. Acad. Sci. USA.

[30] Hopper E., Belinsky M.G., Zeng H., Tosolini A., Testa J.R., Kruh G.D. (2001). Analysis of the structure and expression pattern of MRP7 (ABCC10), a new member of the MRP subfamily. Cancer Lett..

[31] Takayanagi S., Kataoka T., Ohara O., Oishi M., Kuo M.T., Ishikawa T. (2004). Human ATP-binding cassette transporter ABCC10: Expression profile and p53-dependent upregulation. J. Exp. Ther. Oncol..

[32] Hopper-Borge E.A., Churchill T., Paulose C., Nicolas E., Jacobs J.D., Ngo O., Kuang Y., Grinberg A., Westphal H., Chen Z.S. (2011). Contribution of Abcc10 (Mrp7) to *in vivo* paclitaxel resistance as assessed in Abcc10(−/−) Mice. Cancer Res..

[33] Chen Z.S., Hopper-Borge E., Belinsky M.G., Shchaveleva I., Kotova E., Kruh G.D. (2003). Characterization of the transport properties of human multidrug resistance protein 7 (MRP7, ABCC10). Mol. Pharmacol..

[34] Bessho Y., Oguri T., Ozasa H., Uemura T., Sakamoto H., Miyazaki M., Maeno K., Sato S., Ueda R. (2009). ABCC10/MRP7 is associated with vinorelbine resistance in Non-small cell lung cancer. Oncol. Rep..

[35] Naramoto H., Uematsu T., Uchihashi T., Doto R., Matsuura T., Usui Y., Uematsu S., Li X., Takahashi M., Yamaoka M. (2007). Multidrug resistance-Associated protein 7 expression is involved in cross-resistance to docetaxel in salivary gland adenocarcinoma cell lines. Int. J. Oncol..

[36] Dabrowska M., Sirotnak F.M. (2004). Regulation of transcription of the human MRP7 gene: Characteristics of the basal promoter and identification of tumor-derived transcripts encoding additional 5' end heterogeneity. Gene.

[37] Robey R.W., Polgar O., Deeken J., To K.W., Bates S.E. (2007). ABCG2: Determining its relevance in clinical drug resistance. Cancer Metastasis Rev..

[38] Doyle L.A., Yang W., Abruzzo L.V., Krogmann T., Gao Y., Rishi A.K., Ross D.D. (1998). A multidrug resistance transporter from human MCF-7 breast cancer cells. Proc. Natl. Acad. Sci. USA.

[39] Miyake K., Mickley L., Litman T., Zhan Z., Robey R., Cristensen B., Brangi M., Greenberger L., Dean M., Fojo T. (1999). Molecular cloning of cDNAs which are highly overexpressed in mitoxantrone-resistant cells: Demonstration of homology to ABC transport genes. Cancer Res..

[40] Litman T., Brangi M., Hudson E., Fetsch P., Abati A., Ross D.D., Miyake K., Resau J.H., Bates S.E. (2000). The multidrug-resistant phenotype associated with overexpression of the new ABC half-transporter, MXR (ABCG2). J. Cell. Sci..

[41] Maliepaard M., van Gastelen M.A., Tohgo A., Hausheer F.H., van Waardenburg R.C., de Jong L.A., Pluim D., Beijnen J.H., Schellens J.H. (2001). Circumvention of breast cancer resistance protein (BCRP)-mediated resistance to camptothecins *in vitro* using non-substrate drugs or the BCRP inhibitor GF120918. Clin. Cancer Res..

[42] Schinkel A.H., Jonker J.W. (2003). Mammalian drug efflux transporters of the ATP binding cassette (ABC) family: An overview. Adv. Drug Deliv. Rev..

[43] Merino G., Alvarez A.I., Pulido M.M., Molina A.J., Schinkel A.H., Prieto J.G. (2006). Breast cancer resistance protein (BCRP/ABCG2) transports fluoroquinolone antibiotics and affects their oral availability, pharmacokinetics, and milk secretion. Drug Metab. Dispos..

[44] Ejendal K.F., Hrycyna C.A. (2002). Multidrug resistance and cancer: The role of the human ABC transporter ABCG2. Curr. Protein Pept. Sci..

[45] Krishnamurthy P., Schuetz J.D. (2006). Role of ABCG2/BCRP in biology and medicine. Annu. Rev. Pharmacol. Toxicol..

[46] Sharom F.J., Yu X., Lu P., Liu R., Chu J.W., Szabo K., Muller M., Hose C.D., Monks A., Varadi A. (1999). Interaction of the P-Glycoprotein multidrug transporter (MDR1) with high affinity peptide chemosensitizers in isolated membranes, reconstituted systems, and intact cells. Biochem. Pharmacol..

[47] Nie S., Xing Y., Kim G.J., Simons J.W. (2007). Nanotechnology applications in cancer. Annu. Rev. Biomed. Eng..

[48] Basu S., Chaudhuri P., Sengupta S. (2009). Targeting oncogenic signaling pathways by exploiting nanotechnology. Cell Cycle.

[49] Xue X., Liang X.J. (2012). Overcoming drug efflux-based multidrug resistance in cancer with nanotechnology. Chin. J. Cancer.

[50] Shi Z., Peng X.X., Kim I.W., Shukla S., Si Q.S., Robey R.W., Bates S.E., Shen T., Ashby C.R., Fu L.W. (2007). Erlotinib (Tarceva, OSI-774) antagonizes ATP-binding cassette subfamily B member 1 and ATP-binding cassette subfamily G member 2-mediated drug resistance. Cancer Res..

[51] Ludwig J.A., Szakacs G., Martin S.E., Chu B.F., Cardarelli C., Sauna Z.E., Caplen N.J., Fales H.M., Ambudkar S.V., Weinstein J.N. (2006). Selective toxicity of NSC73306 in MDR1-positive cells as a new strategy to circumvent multidrug resistance in cancer. Cancer Res..

[52] Krause D.S., van Etten R.A. (2005). Tyrosine kinases as targets for cancer therapy. N. Engl. J. Med..

[53] Faivre S., Djelloul S., Raymond E. (2006). New paradigms in anticancer therapy: Targeting multiple signaling pathways with kinase inhibitors. Semin. Oncol..

[54] Luke J.J., Hodi F.S. (2012). Vemurafenib and BRAF inhibition: A new class of treatment for metastatic melanoma. Clin. Cancer Res..

[55] Vispute S.G., Chen J.J., Sun Y.L., Sodani K.S., Singh S., Pan Y., Talele T., Ashby C.R., Chen Z.S. (2013). Vemurafenib (plx4032, zelboraf^®^), a BRAF inhibitor, modulates ABCB1-, ABCG2-, and ABCC10-mediated multidrug resistance. J. Cancer Res. Updates.

[56] Dai C.L., Tiwari A.K., Wu C.P., Su X.D., Wang S.R., Liu D.G., Ashby C.R., Huang Y., Robey R.W., Liang Y.J. (2008). Lapatinib (Tykerb, GW572016) reverses multidrug resistance in cancer cells by inhibiting the activity of ATP-binding cassette subfamily B member 1 and G member 2. Cancer Res..

[57] Shen T., Kuang Y.H., Ashby C.R., Lei Y., Chen A., Zhou Y., Chen X., Tiwari A.K., Hopper-Borge E., Ouyang J. (2009). Imatinib and nilotinib reverse multidrug resistance in cancer cells by inhibiting the efflux activity of the MRP7 (ABCC10). PLoS One.

[58] Tiwari A.K., Sodani K., Wang S.R., Kuang Y.H., Ashby C.R., Chen X., Chen Z.S. (2009). Nilotinib (AMN107, Tasigna) reverses multidrug resistance by inhibiting the activity of the ABCB1/Pgp and ABCG2/BCRP/MXR transporters. Biochem. Pharmacol..

[59] Tiwari A.K., Sodani K., Dai C.L., Abuznait A.H., Singh S., Xiao Z.J., Patel A., Talele T.T., Fu L., Kaddoumi A. (2013). Nilotinib potentiates anticancer drug sensitivity in murine ABCB1-, ABCG2-, and ABCC10-multidrug resistance xenograft models. Cancer Lett..

[60] Kathawala R.J., Sodani K., Chen K., Patel A., Abuznait A.H., Anreddy N., Sun Y.L., Kaddoumi A., Ashby C.R., Chen Z.S. (2014). Masitinib antagonizes ATP-binding cassette subfamily C member 10-mediated paclitaxel resistance: A preclinical study. Mol. Cancer. Ther..

[61] Sodani K., Patel A., Anreddy N., Singh S., Yang D.H., Kathawala R.J., Kumar P., Talele T.T., Chen Z.S. (2014). Telatinib reverses chemotherapeutic multidrug resistance mediated by ABCG2 efflux transporter *in vitro* and *in vivo*. Biochem. Pharmacol..

[62] Zhang H., Kathawala R.J., Wang Y.J., Zhang Y.K., Patel A., Shukla S., Robey R.W., Talele T.T., Ashby C.R., Ambudkar S.V. (2014). Linsitinib (OSI-906) antagonizes ATP-binding cassette subfamily G member 2 and subfamily C member 10-mediated drug resistance. Int. J. Biochem. Cell Biol..

[63] Zhang H., Wang Y.J., Zhang Y.K., Wang D.S., Kathawala R.J., Patel A., Talele T.T., Chen Z.S., Fu L.W. (2014). AST1306, a potent EGFR inhibitor, antagonizes ATP-binding cassette subfamily G member 2-mediated multidrug resistance. Cancer Lett..

[64] Zhang H., Zhang Y.K., Wang Y.J., Kathawala R.J., Patel A., Zhu H., Sodani K., Talele T.T., Ambudkar S.V., Chen Z.S. (2014). WHI-P154 enhances the chemotherapeutic effect of anticancer agents in ABCG2-overexpressing cells. Cancer Sci..

[65] Wang Y.J., Kathawala R.J., Zhang Y.K., Patel A., Kumar P., Shukla S., Fung K.L., Ambudkar S.V., Talele T.T., Chen Z.S. (2014). Motesanib (AMG706), a potent multikinase inhibitor, antagonizes multidrug resistance by inhibiting the efflux activity of the ABCB1. Biochem. Pharmacol..

[66] Aller S.G., Yu J., Ward A., Weng Y., Chittaboina S., Zhuo R., Harrell P.M., Trinh Y.T., Zhang Q., Urbatsch I.L. (2009). Structure of P-Glycoprotein reveals a molecular basis for poly-specific drug binding. Science.

[67] Nicolle E., Boumendjel A., Macalou S., Genoux E., Ahmed-Belkacem A., Carrupt P.A., di Pietro A. (2009). QSAR analysis and molecular modeling of ABCG2-specific inhibitors. Adv. Drug Deliv. Rev..

[68] Crivori P., Reinach B., Pezzetta D., Poggesi I. (2006). Computational models for identifying potential P-Glycoprotein substrates and inhibitors. Mol. Pharm..

[69] Pajeva I.K., Globisch C., Wiese M. (2009). Combined pharmacophore modeling, docking, and 3D QSAR studies of ABCB1 and ABCC1 transporter inhibitors. ChemMedChem.

[70] Xie H., Lin L., Tong L., Jiang Y., Zheng M., Chen Z., Jiang X., Zhang X., Ren X., Qu W. (2011). AST1306, a novel irreversible inhibitor of the epidermal growth factor receptor 1 and 2, exhibits antitumor activity both *in vitro* and *in vivo*. PLoS One.

[71] Zhang J., Cao J., Li J., Zhang Y., Chen Z., Peng W., Sun S., Zhao N., Wang J., Zhong D. (2014). A phase I study of AST1306, a novel irreversible EGFR and HER2 kinase inhibitor, in patients with advanced solid tumors. J. Hematol. Oncol..

[72] Normanno N., de Luca A., Bianco C., Strizzi L., Mancino M., Maiello M.R., Carotenuto A., de Feo G., Caponigro F., Salomon D.S. (2006). Epidermal growth factor receptor (EGFR) signaling in cancer. Gene.

[73] Mulvihill M.J., Cooke A., Rosenfeld-Franklin M., Buck E., Foreman K., Landfair D., O’Connor M., Pirritt C., Sun Y., Yao Y. (2009). Discovery of OSI-906: A selective and orally efficacious dual inhibitor of the IGF-1 receptor and insulin receptor. Future Med. Chem..

[74] McKinley E.T., Bugaj J.E., Zhao P., Guleryuz S., Mantis C., Gokhale P.C., Wild R., Manning H.C. (2011). 18FDG-PET Predicts pharmacodynamic response to OSI-906, a dual IGF-1R/IR inhibitor, in preclinical mouse models of lung cancer. Clin. Cancer Res..

[75] Le Cesne A., Blay J.Y., Bui B.N., Bouche O., Adenis A., Domont J., Cioffi A., Ray-Coquard I., Lassau N., Bonvalot S. (2010). Phase II study of oral masitinib mesilate in imatinib-naive patients with locally advanced or metastatic gastro-intestinal stromal tumour (GIST). Eur. J. Cancer.

[76] Mitry E., Hammel P., Deplanque G., Mornex F., Levy P., Seitz J.F., Moussy A., Kinet J.P., Hermine O., Rougier P. (2010). Safety and activity of masitinib in combination with gemcitabine in patients with advanced pancreatic cancer. Cancer Chemother. Pharmacol..

[77] Georgin-Lavialle S., Lhermitte L., Suarez F., Yang Y., Letard S., Hanssens K., Feger F., Renand A., Brouze C., Canioni D. (2012). Mast cell leukemia: Identification of a new C-Kit mutation, dup(501–502), and response to masitinib, a C-Kit tyrosine kinase inhibitor. Eur. J. Haematol..

[78] Kathawala R.J., Chen J.J., Zhang Y.K., Wang Y.J., Patel A., Wang D.S., Talele T.T., Ashby C.R., Chen Z.S. (2014). Masitinib antagonizes ATP-binding cassette subfamily G member 2-mediated multidrug resistance. Int. J. Oncol..

[79] Polverino A., Coxon A., Starnes C., Diaz Z., DeMelfi T., Wang L., Bready J., Estrada J., Cattley R., Kaufman S. (2006). AMG 706, an oral, multikinase inhibitor that selectively targets vascular endothelial growth factor, platelet-derived growth factor, and kit receptors, potently inhibits angiogenesis and induces regression in tumor xenografts. Cancer Res..

[80] Gora-Tybor J., Robak T. (2008). Targeted drugs in chronic myeloid leukemia. Curr. Med. Chem..

[81] Shah N.P., Nicoll J.M., Nagar B., Gorre M.E., Paquette R.L., Kuriyan J., Sawyers C.L. (2002). Multiple BCR-ABL kinase domain mutations confer polyclonal resistance to the tyrosine kinase inhibitor imatinib (STI571) in chronic phase and blast crisis chronic myeloid leukemia. Cancer Cell.

[82] Langenberg M.H., Witteveen P.O., Roodhart J.M., Verheul H.M., Mergui-Roelvink M., van der Sar J., Brendel E., Laferriere N., Schellens J.H., Voest E.E. (2010). Phase I evaluation of telatinib, a vascular endothelial growth factor receptor tyrosine kinase inhibitor, in combination with irinotecan and capecitabine in patients with advanced solid tumors. Clin. Cancer Res..

[83] Sudbeck E.A., Ghosh S., Liu X.P., Zheng Y., Myers D.E., Uckun F.M. (2001). Tyrosine kinase inhibitors against EGF receptor-positive malignancies. Methods Mol. Biol..

[84] Marzec M., Kasprzycka M., Ptasznik A., Wlodarski P., Zhang Q., Odum N., Wasik M.A. (2005). Inhibition of ALK enzymatic activity in T-cell lymphoma cells induces apoptosis and suppresses proliferation and STAT3 phosphorylation independently of Jak3. Lab. Invest..

[85] Stewart C.F., Leggas M., Schuetz J.D., Panetta J.C., Cheshire P.J., Peterson J., Daw N., Jenkins J.J., Gilbertson R., Germain G.S. (2004). Gefitinib enhances the antitumor activity and oral bioavailability of irinotecan in mice. Cancer Res..

[86] Nakamura Y., Oka M., Soda H., Shiozawa K., Yoshikawa M., Itoh A., Ikegami Y., Tsurutani J., Nakatomi K., Kitazaki T. (2005). Gefitinib (“Iressa”, ZD1839), an epidermal growth factor receptor tyrosine kinase inhibitor, reverses breast cancer resistance protein/ABCG2-mediated drug resistance. Cancer Res..

[87] Chen K.G., Sikic B.I. (2012). Molecular pathways: Regulation and therapeutic implications of multidrug resistance. Clin. Cancer Res..

[88] Sikic B.I., Fisher G.A., Lum B.L., Halsey J., Beketic-Oreskovic L., Chen G. (1997). Modulation and prevention of multidrug resistance by inhibitors of P-Glycoprotein. Cancer Chemother. Pharmacol..

[89] Yahanda A.M., Alder K.M., Fisher G.A., Brophy N.A., Halsey J., Hardy R.I., Gosland M.P., Lum B.L., Sikic B.I. (1992). Phase I trial of etoposide with cyclosporine as a modulator of multidrug resistance. J. Clin. Oncol..

[90] Lum B.L., Kaubisch S., Yahanda A.M., Adler K.M., Jew L., Ehsan M.N., Brophy N.A., Halsey J., Gosland M.P., Sikic B.I. (1992). Alteration of etoposide pharmacokinetics and pharmacodynamics by cyclosporine in a phase I trial to modulate multidrug resistance. J. Clin. Oncol..

[91] Bartlett N.L., Lum B.L., Fisher G.A., Brophy N.A., Ehsan M.N., Halsey J., Sikic B.I. (1994). Phase I trial of doxorubicin with cyclosporine as a modulator of multidrug resistance. J. Clin. Oncol..

[92] Advani R., Fisher G.A., Lum B.L., Hausdorff J., Halsey J., Litchman M., Sikic B.I. (2001). A phase I trial of doxorubicin, paclitaxel, and valspodar (PSC 833), a modulator of multidrug resistance. Clin. Cancer Res..

[93] Baer M.R., George S.L., Dodge R.K., OʼLoughlin K.L., Minderman H., Caligiuri M.A., Anastasi J., Powell B.L., Kolitz J.E., Schiffer C.A. (2002). Phase 3 study of the multidrug resistance modulator PSC-833 in previously untreated patients 60 years of age and older with acute myeloid leukemia: Cancer and leukemia group B study 9720. Blood.

[94] List A.F., Kopecky K.J., Willman C.L., Head D.R., Persons D.L., Slovak M.L., Dorr R., Karanes C., Hynes H.E., Doroshow J.H. (2001). Benefit of cyclosporine modulation of drug resistance in patients with poor-risk acute myeloid leukemia: A southwest oncology group study. Blood.

[95] Liu Yin J.A., Wheatley K., Rees J.K., Burnett A.K., UK MRC Adult Leukemia Working Party (2001). Comparison of “Sequential” versus “Standard” chemotherapy as re-induction treatment, with or without cyclosporine, in refractory/relapsed acute myeloid leukaemia (AML): Results of the UK medical research council AML-R trial. Br. J. Haematol..

[96] Cripe L.D., Uno H., Paietta E.M., Litzow M.R., Ketterling R.P., Bennett J.M., Rowe J.M., Lazarus H.M., Luger S., Tallman M.S. (2010). Zosuquidar, a novel modulator of P-Glycoprotein, does not improve the outcome of older patients with newly diagnosed acute myeloid leukemia: A randomized, Placebo-controlled trial of the eastern Cooperative Oncology Group 3999. Blood.

[97] Qadir M., O’Loughlin K.L., Fricke S.M., Williamson N.A., Greco W.R., Minderman H., Baer M.R. (2005). Cyclosporin A is a broad-spectrum multidrug resistance modulator. Clin. Cancer Res..

[98] Matsubara H., Watanabe M., Imai T., Yui Y., Mizushima Y., Hiraumi Y., Kamitsuji Y., Watanabe K., Nishijo K., Toguchida J. (2009). Involvement of extracellular signal-regulated kinase activation in human osteosarcoma cell resistance to the histone deacetylase inhibitor FK228 [(1S,4S,7Z,10S,16E,21R)-7-Ethylidene-4,21-Bis(Propan-2-Yl)-2-Oxa-12,13-Dithia-5,8,20,23-Tetraazabicyclo[8.7.6]Tricos-16-Ene-3,6,9,19,22-Pentone. J. Pharmacol. Exp. Ther..

[99] Tabe Y., Konopleva M., Contractor R., Munsell M., Schober W.D., Jin L., Tsutsumi-Ishii Y., Nagaoka I., Igari J., Andreeff M. (2006). Up-regulation of MDR1 and induction of doxorubicin resistance by histone deacetylase inhibitor depsipeptide (FK228) and ATRA in acute promyelocytic leukemia cells. Blood.

[100] Van den Heuvel-Eibrink M.M., Wiemer E.A., Prins A., Meijerink J.P., Vossebeld P.J., van der Holt B., Pieters R., Sonneveld P. (2002). Increased expression of the breast cancer resistance protein (BCRP) in relapsed or refractory acute myeloid leukemia (AML). Leukemia.

[101] Tang S.C., Lagas J.S., Lankheet N.A., Poller B., Hillebrand M.J., Rosing H., Beijnen J.H., Schinkel A.H. (2012). Brain accumulation of sunitinib is restricted by P-Glycoprotein (ABCB1) and breast cancer resistance protein (ABCG2) and can be enhanced by oral elacridar and sunitinib coadministration. Int. J. Cancer.

[102] Plasschaert S.L., van der Kolk D.M., de Bont E.S., Kamps W.A., Morisaki K., Bates S.E., Scheffer G.L., Scheper R.J., Vellenga E., de Vries E.G. (2003). The role of breast cancer resistance protein in acute lymphoblastic leukemia. Clin. Cancer Res..

[103] Sauerbrey A., Sell W., Steinbach D., Voigt A., Zintl F. (2002). Expression of the BCRP gene (ABCG2/MXR/ABCP) in childhood acute lymphoblastic leukaemia. Br. J. Haematol..

[104] Jordanides N.E., Jorgensen H.G., Holyoake T.L., Mountford J.C. (2006). Functional ABCG2 is overexpressed on primary CML CD34+ cells and is Inhibited by imatinib mesylate. Blood.

[105] Kim J.E., Singh R.R., Cho-Vega J.H., Drakos E., Davuluri Y., Khokhar F.A., Fayad L., Medeiros L.J., Vega F. (2009). Sonic hedgehog signaling proteins and ATP-binding cassette G2 are aberrantly expressed in diffuse large B-Cell lymphoma. Mod. Pathol..

[106] Szczuraszek K., Materna V., Halon A., Mazur G., Wrobel T., Kuliczkowski K., Maciejczyk A., Zabel M., Drag M., Dietel M. (2009). Positive correlation between cyclooxygenase-2 and ABC-transporter expression in non-hodgkin’s lymphomas. Oncol. Rep..

[107] Kim Y.H., Ishii G., Goto K., Ota S., Kubota K., Murata Y., Mishima M., Saijo N., Nishiwaki Y., Ochiai A. (2009). Expression of breast cancer resistance protein is associated with a poor clinical outcome in patients with small-cell lung cancer. Lung Cancer.

[108] Rijavec M., Silar M., Triller N., Kern I., Cegovnik U., Kosnik M., Korosec P. (2011). Expressions of topoisomerase IIalpha and BCRP in metastatic cells are associated with overall survival in small cell lung cancer patients. Pathol. Oncol. Res..

[109] Yamada A., Ishikawa T., Ota I., Kimura M., Shimizu D., Tanabe M., Chishima T., Sasaki T., Ichikawa Y., Morita S. (2013). High expression of ATP-binding cassette transporter ABCC11 in breast tumors is associated with aggressive subtypes and low disease-free survival. Breast Cancer Res. Treat..

[110] Hlavata I., Mohelnikova-Duchonova B., Vaclavikova R., Liska V., Pitule P., Novak P., Bruha J., Vycital O., Holubec L., Treska V. (2012). The role of ABC transporters in progression and clinical outcome of colorectal cancer. Mutagenesis.

[111] Kunicka T., Soucek P. (2014). Importance of ABCC1 for cancer therapy and prognosis. Drug Metab. Rev..

[112] Young L.C., Campling B.G., Cole S.P., Deeley R.G., Gerlach J.H. (2001). Multidrug resistance proteins MRP3, MRP1, and MRP2 in lung cancer: Correlation of protein levels with drug response and messenger RNA levels. Clin. Cancer Res..

[113] Zalcberg J., Hu X.F., Slater A., Parisot J., El-Osta S., Kantharidis P., Chou S.T., Parkin J.D. (2000). MRP1 Not MDR1 Gene expression is the predominant mechanism of acquired multidrug resistance in two prostate carcinoma cell lines. Prostate Cancer Prostatic Dis..

[114] Chauhan P.S., Bhushan B., Singh L.C., Mishra A.K., Saluja S., Mittal V., Gupta D.K., Kapur S. (2012). Expression of genes related to multiple drug resistance and apoptosis in acute leukemia: Response to induction chemotherapy. Exp. Mol. Pathol..

[115] Cui Y., Konig J., Buchholz J.K., Spring H., Leier I., Keppler D. (1999). Drug resistance and ATP-dependent conjugate transport mediated by the apical multidrug resistance protein, MRP2, permanently expressed in human and canine cells. Mol. Pharmacol..

[116] Huisman M.T., Chhatta A.A., van Tellingen O., Beijnen J.H., Schinkel A.H. (2005). MRP2 (ABCC2) transports taxanes and confers paclitaxel resistance and both processes are stimulated by probenecid. Int. J. Cancer.

[117] Guminski A.D., Balleine R.L., Chiew Y.E., Webster L.R., Tapner M., Farrell G.C., Harnett P.R., Defazio A. (2006). MRP2 (ABCC2) and cisplatin sensitivity in hepatocytes and human ovarian carcinoma. Gynecol. Oncol..

[118] Zelcer N., Saeki T., Reid G., Beijnen J.H., Borst P. (2001). Characterization of drug transport by the human multidrug resistance protein 3 (ABCC3). J. Biol. Chem..

[119] Zeng H., Chen Z.S., Belinsky M.G., Rea P.A., Kruh G.D. (2001). Transport of methotrexate (MTX) and folates by multidrug resistance protein (MRP) 3 and MRP1: Effect of polyglutamylation on MTX transport. Cancer Res..

[120] Chen Z.S., Lee K., Kruh G.D. (2001). Transport of cyclic nucleotides and estradiol 17-Beta-D-Glucuronide by multidrug resistance protein 4. Resistance to 6-mercaptopurine and 6-thioguanine. J. Biol. Chem..

[121] Tian Q., Zhang J., Tan T.M., Chan E., Duan W., Chan S.Y., Boelsterli U.A., Ho P.C., Yang H., Bian J.S. (2005). Human multidrug resistance associated protein 4 confers resistance to camptothecins. Pharm. Res..

[122] Pratt S., Shepard R.L., Kandasamy R.A., Johnston P.A., Perry W., Dantzig A.H. (2005). The multidrug resistance protein 5 (ABCC5) confers resistance to 5-fluorouracil and transports its monophosphorylated metabolites. Mol. Cancer. Ther..

[123] Belinsky M.G., Chen Z.S., Shchaveleva I., Zeng H., Kruh G.D. (2002). Characterization of the drug resistance and transport properties of multidrug resistance protein 6 (MRP6, ABCC6). Cancer Res..

[124] Wang P., Zhang Z., Gao K., Deng Y., Zhao J., Liu B., Li X. (2009). Expression and clinical significance of ABCC10 in the patients with non-small cell lung cancer (in Chinese). Zhongguo Fei Ai Za Zhi.

[125] Hu S., Niu H., Inaba H., Orwick S., Rose C., Panetta J.C., Yang S., Pounds S., Fan Y., Calabrese C. (2011). Activity of the multikinase inhibitor sorafenib in combination with cytarabine in acute myeloid leukemia. J. Natl. Cancer Inst..

[126] Walle T., Walle U.K., Kumar G.N., Bhalla K.N. (1995). Taxol metabolism and disposition in cancer patients. Drug Metab. Dispos..

[127] Sparreboom A., van Asperen J., Mayer U., Schinkel A.H., Smit J.W., Meijer D.K., Borst P., Nooijen W.J., Beijnen J.H., van Tellingen O. (1997). Limited oral bioavailability and active epithelial excretion of paclitaxel (Taxol) caused by P-Glycoprotein in the intestine. Proc. Natl. Acad. Sci. USA.

[128] Green H., Soderkvist P., Rosenberg P., Horvath G., Peterson C. (2008). ABCB1 G1199A polymorphism and ovarian cancer response to paclitaxel. J. Pharm. Sci..

[129] Green H., Soderkvist P., Rosenberg P., Horvath G., Peterson C. (2006). Mdr-1 single nucleotide polymorphisms in ovarian cancer tissue: G2677T/A correlates with response to paclitaxel chemotherapy. Clin. Cancer Res..

[130] Sissung T.M., Mross K., Steinberg S.M., Behringer D., Figg W.D., Sparreboom A., Mielke S. (2006). Association of ABCB1 genotypes with paclitaxel-mediated peripheral neuropathy and neutropenia. Eur. J. Cancer.

[131] Nakajima M., Fujiki Y., Kyo S., Kanaya T., Nakamura M., Maida Y., Tanaka M., Inoue M., Yokoi T. (2005). Pharmacokinetics of paclitaxel in ovarian cancer patients and genetic polymorphisms of CYP2C8, CYP3A4, and MDR1. J. Clin. Pharmacol..

[132] Henningsson A., Marsh S., Loos W.J., Karlsson M.O., Garsa A., Mross K., Mielke S., Vigano L., Locatelli A., Verweij J. (2005). Association of CYP2C8, CYP3A4, CYP3A5, and ABCB1 polymorphisms with the pharmacokinetics of paclitaxel. Clin. Cancer Res..

[133] Grimm C., Polterauer S., Zeillinger R., Tong D., Heinze G., Wolf A., Natter C., Reinthaller A., Hefler L.A. (2010). Two multidrug-resistance (ABCB1) gene polymorphisms as prognostic parameters in women with ovarian cancer. Anticancer Res..

[134] Marsh S., Paul J., King C.R., Gifford G., McLeod H.L., Brown R. (2007). Pharmacogenetic assessment of toxicity and outcome after platinum plus taxane chemotherapy in ovarian cancer: The Scottish randomised trial in ovarian cancer. J. Clin. Oncol..

[135] Wojnowski L., Kulle B., Schirmer M., Schluter G., Schmidt A., Rosenberger A., Vonhof S., Bickeboller H., Toliat M.R., Suk E.K. (2005). NAD(P)H oxidase and multidrug resistance protein genetic polymorphisms are associated with doxorubicin-induced cardiotoxicity. Circulation.

[136] Colombo S., Soranzo N., Rotger M., Sprenger R., Bleiber G., Furrer H., Buclin T., Goldstein D., Decosterd L., Telenti A. (2005). Influence of ABCB1, ABCC1, ABCC2, and ABCG2 haplotypes on the cellular exposure of nelfinavir *in vivo*. Pharmacogenet. Genomics.

[137] Kartenbeck J., Leuschner U., Mayer R., Keppler D. (1996). Absence of the canalicular isoform of the MRP gene-encoded conjugate export pump from the hepatocytes in dubin-johnson syndrome. Hepatology.

[138] Gradhand U., Kim R.B. (2008). Pharmacogenomics of MRP transporters (ABCC1–5) and BCRP (ABCG2). Drug Metab. Rev..

[139] Kato T., Hamada A., Mori S., Saito H. (2012). Genetic polymorphisms in metabolic and cellular transport pathway of methotrexate impact clinical outcome of methotrexate monotherapy in Japanese patients with rheumatoid arthritis. Drug Metab. Pharmacokinet..

[140] Pushpakom S.P., Liptrott N.J., Rodriguez-Novoa S., Labarga P., Soriano V., Albalater M., Hopper-Borge E., Bonora S., di Perri G., Back D.J. (2011). Genetic variants of ABCC10, a novel tenofovir transporter, are associated with kidney tubular dysfunction. J. Infect. Dis..

[141] Liptrott N.J., Pushpakom S., Wyen C., Fatkenheuer G., Hoffmann C., Mauss S., Knechten H., Brockmeyer N.H., Hopper-Borge E., Siccardi M. (2012). Association of ABCC10 polymorphisms with nevirapine plasma concentrations in the german competence network for HIV/AIDS. Pharmacogenet. Genomics.

[142] Ciccacci C., di Fusco D., Marazzi M.C., Liotta G., Palombi L., Novelli G., Borgiani P. (2013). ABCC10 rs2125739 polymorphism and nevirapine-induced hepatotoxicity: Lack of association in a population from mozambique. Pharmacogenet. Genomics.

[143] Cusatis G., Sparreboom A. (2008). Pharmacogenomic importance of ABCG2. Pharmacogenomics.

[144] Cusatis G., Gregorc V., Li J., Spreafico A., Ingersoll R.G., Verweij J., Ludovini V., Villa E., Hidalgo M., Sparreboom A. (2006). Pharmacogenetics of ABCG2 and adverse reactions to gefitinib. J. Natl. Cancer Inst..

[145] Zamboni W.C., Ramanathan R.K., McLeod H.L., Mani S., Potter D.M., Strychor S., Maruca L.J., King C.R., Jung L.L., Parise R.A. (2006). Disposition of 9-nitrocamptothecin and its 9-aminocamptothecin metabolite in relation to ABC transporter genotypes. Invest. New Drugs.

[146] Sparreboom A., Gelderblom H., Marsh S., Ahluwalia R., Obach R., Principe P., Twelves C., Verweij J., McLeod H.L. (2004). Diflomotecan pharmacokinetics in relation to ABCG2 421C > A genotype. Clin. Pharmacol. Ther..

[147] Zhou Q., Sparreboom A., Tan E.H., Cheung Y.B., Lee A., Poon D., Lee E.J., Chowbay B. (2005). Pharmacogenetic profiling across the irinotecan pathway in asian patients with cancer. Br. J. Clin. Pharmacol..

[148] Zhang W., Yu B.N., He Y.J., Fan L., Li Q., Liu Z.Q., Wang A., Liu Y.L., Tan Z.R., Jiang F. (2006). Role of BCRP 421C > A polymorphism on rosuvastatin pharmacokinetics in healthy Chinese males. Clin. Chim. Acta.

[149] Urquhart B.L., Ware J.A., Tirona R.G., Ho R.H., Leake B.F., Schwarz U.I., Zaher H., Palandra J., Gregor J.C., Dresser G.K. (2008). Breast cancer resistance protein (ABCG2) and drug disposition: Intestinal expression, polymorphisms and sulfasalazine as an *in vivo* probe. Pharmacogenet. Genomics.

[150] Yamasaki Y., Ieiri I., Kusuhara H., Sasaki T., Kimura M., Tabuchi H., Ando Y., Irie S., Ware J., Nakai Y. (2008). Pharmacogenetic characterization of sulfasalazine disposition based on NAT2 and ABCG2 (BCRP) gene polymorphisms in humans. Clin. Pharmacol. Ther..

[151] Sparreboom A., Loos W.J., Burger H., Sissung T.M., Verweij J., Figg W.D., Nooter K., Gelderblom H. (2005). Effect of ABCG2 genotype on the oral bioavailability of topotecan. Cancer Biol. Ther..

[152] Lal S., Wong Z.W., Sandanaraj E., Xiang X., Ang P.C., Lee E.J., Chowbay B. (2008). Influence of ABCB1 and ABCG2 polymorphisms on doxorubicin disposition in Asian breast cancer patients. Cancer Sci..

[153] Gardner E.R., Burger H., van Schaik R.H., van Oosterom A.T., de Bruijn E.A., Guetens G., Prenen H., de Jong F.A., Baker S.D., Bates S.E. (2006). Association of enzyme and transporter genotypes with the pharmacokinetics of imatinib. Clin. Pharmacol. Ther..

[154] Ieiri I., Suwannakul S., Maeda K., Uchimaru H., Hashimoto K., Kimura M., Fujino H., Hirano M., Kusuhara H., Irie S. (2007). SLCO1B1 (OATP1B1, an uptake transporter) and ABCG2 (BCRP, an efflux transporter) variant alleles and pharmacokinetics of pitavastatin in healthy volunteers. Clin. Pharmacol. Ther..

[155] Yoshioka S., Katayama K., Okawa C., Takahashi S., Tsukahara S., Mitsuhashi J., Sugimoto Y. (2007). The identification of two germ-line mutations in the human breast cancer resistance protein gene that result in the expression of a low/non-functional protein. Pharm. Res..

[156] Rudin C.M., Liu W., Desai A., Karrison T., Jiang X., Janisch L., Das S., Ramirez J., Poonkuzhali B., Schuetz E. (2008). Pharmacogenomic and pharmacokinetic determinants of erlotinib toxicity. J. Clin. Oncol..

[157] Poonkuzhali B., Lamba J., Strom S., Sparreboom A., Thummel K., Watkins P., Schuetz E. (2008). Association of breast cancer resistance protein/ABCG2 phenotypes and novel promoter and intron 1 single nucleotide polymorphisms. Drug Metab. Dispos..

[158] Innocenti F., Kroetz D.L., Schuetz E., Dolan M.E., Ramirez J., Relling M., Chen P., Das S., Rosner G.L., Ratain M.J. (2009). Comprehensive pharmacogenetic analysis of irinotecan neutropenia and pharmacokinetics. J. Clin. Oncol..

[159] Cecchin E., D’Andrea M., Lonardi S., Zanusso C., Pella N., Errante D., de Mattia E., Polesel J., Innocenti F., Toffoli G. (2013). A prospective validation pharmacogenomic study in the adjuvant setting of colorectal cancer patients treated with the 5-Fluorouracil/Leucovorin/Oxaliplatin (FOLFOX4) regimen. Pharmacogenomics J..

